# Reply to Comment on “Jilkova, Z.M.; et al. Predictive Factors for Response to PD-1/PD-L1 Checkpoint Inhibition in the Field of Hepatocellular Carcinoma: Current Status and Challenges” *Cancers* 2019, *11*, 1554

**DOI:** 10.3390/cancers12092673

**Published:** 2020-09-18

**Authors:** Zuzana Macek Jilkova, Caroline Aspord, Thomas Decaens

**Affiliations:** 1 Université Grenoble Alpes, 38000 Grenoble, France; Caroline.Aspord@efs.sante.fr (C.A.); tdecaens@chu-grenoble.fr (T.D.); 2 Institute for Advanced Biosciences, Research Center UGA/Inserm U 1209/CNRS 5309, 38700 La Tronche, France; 3 Service d’hépato-gastroentérologie, Pôle Digidune, CHU Grenoble Alpes, 38700 La Tronche, France; 4 Etablissement Français du Sang Auvergne-Rhône-Alpes, R&D-Laboratory, 38701 Grenoble, France

We appreciate the comments made on our review paper focused on possible predictive factors for responses to PD-1/PD-L1 checkpoint inhibitors in the field of hepatocellular carcinoma [1]. We fully agree that the statement “Certainly, high tumor mutational burden and neoantigen load have been noted to predict the response to immunotherapies, including anti-PD-1 therapy (higher objective response rate and/or prolonged survival) in melanoma, non-small-cell lung carcinoma” is not supported by the given reference of Topalian et al. for the year 2012 [2]. As mentioned in the comment, the correct reference is Goodman et al.’s work from 2017 [3], cited in our original review as reference number 60, where the authors demonstrated a linear correlation between higher tumor mutational burden and better outcome parameters based on 151 immunotherapy-treated patients. In addition, our statement can be supported by a recent meta-analysis from Kim et al. 2019 [4], which focused on the tumor mutational burden and efficacy of immune checkpoint inhibitors in more than 5000 patients with various advanced cancer types. The authors showed that the patients with high tumor mutational burden have significantly higher objective response rates and prolonged survival as compared to patients with low tumor mutational burden undergoing immune checkpoint inhibitor treatment.

Nonetheless, we completely agree that randomized controlled clinical trials are needed to determine the ability of tumor mutational burden to predict responses to PD-1/PD-L1 checkpoint inhibitors, mainly in different tumor types.
